# General Anesthetics Inhibit Erythropoietin Induction under Hypoxic Conditions in the Mouse Brain

**DOI:** 10.1371/journal.pone.0029378

**Published:** 2011-12-27

**Authors:** Tomoharu Tanaka, Shinichi Kai, Tomohiro Koyama, Hiroki Daijo, Takehiko Adachi, Kazuhiko Fukuda, Kiichi Hirota

**Affiliations:** 1 Department of Anesthesia, Kyoto University Hospital, Kyoto, Japan; 2 Department of Anesthesia, Tazuke Kofukai Medical Research Institute, Kitano Hospital, Osaka, Japan; Hôpital Robert Debré, France

## Abstract

**Background:**

Erythropoietin (EPO), originally identified as a hematopoietic growth factor produced in the kidney and fetal liver, is also endogenously expressed in the central nervous system (CNS). EPO in the CNS, mainly produced in astrocytes, is induced under hypoxic conditions in a hypoxia-inducible factor (HIF)-dependent manner and plays a dominant role in neuroprotection and neurogenesis. We investigated the effect of general anesthetics on EPO expression in the mouse brain and primary cultured astrocytes.

**Methodology/Principal Findings:**

BALB/c mice were exposed to 10% oxygen with isoflurane at various concentrations (0.10–1.0%). Expression of EPO mRNA in the brain was studied, and the effects of sevoflurane, halothane, nitrous oxide, pentobarbital, ketamine, and propofol were investigated. In addition, expression of HIF-2α protein was studied by immunoblotting. Hypoxia-induced EPO mRNA expression in the brain was significantly suppressed by isoflurane in a concentration-dependent manner. A similar effect was confirmed for all other general anesthetics. Hypoxia-inducible expression of HIF-2α protein was also significantly suppressed with isoflurane. In the experiments using primary cultured astrocytes, isoflurane, pentobarbital, and ketamine suppressed hypoxia-inducible expression of HIF-2α protein and EPO mRNA.

**Conclusions/Significance:**

Taken together, our results indicate that general anesthetics suppress activation of HIF-2 and inhibit hypoxia-induced EPO upregulation in the mouse brain through a direct effect on astrocytes.

## Introduction

Ischemic and hypoxic insults to the brain during surgery and anesthesia result in life-threatening complications including stroke. These complications occur at the rate of 0.08–0.7% in general surgery and 1.4–3.8% in cardiac surgery [1]. Pharmacologic interventions, including calcium channel blockers, free radical scavengers, and glutamate antagonists, have been introduced to prevent and/or ameliorate stroke [2]. Erythropoietin (EPO) is also recognized as a promising molecule to introduce neuroprotection, and encouraging results have been obtained from clinical trials involving stroke patients [3], [4], [5]. Originally, EPO was widely known as a hematopoietic growth factor produced in the kidney and fetal liver [5]. Further investigations expanded this review by showing that EPO and EPO receptor (EPOR) are present in the human brain and synthesized locally by astrocytes and neurons [6], [7], [8], [9]. It is well documented in both experimental and clinical studies that EPO produced in the brain acts in a paracrine or autocrine manner to provide neuroprotection [10], [11]. Endogenous EPO in the brain is produced in an oxygen tension-dependent manner [12] and reduces brain damage by inhibiting apoptosis [13], suppressing glutamate release [14], and reducing the production of proinflammatory cytokines [15].

Hypoxia-induced EPO upregulation in the brain is regulated mainly by hypoxia-inducible factor (HIF)-1 and HIF-2 [16]. HIF is a transcriptional factor that acts as a key regulator in cells exposed to low oxygen [17], [18]. In fact, HIF-1 was originally cloned as a transcription factor responsible for hypoxia-induced EPO expression [17]. HIF is a heterodimeric DNA-binding complex composed of two basic helix-loop-helix proteins of the PER-ARNT-SIM (PAS) family: the constitutive non-oxygen-responsive subunit HIF-1β (also termed as the aryl hydrocarbon receptor nuclear translocator: ARNT) and one of either of the hypoxia-inducible α-subunits HIF-1α or HIF-2α [19], [20]. HIF-α proteins are rapidly degraded in normoxia but highly induced by hypoxia [19], [20], [21]. HIF-1α and HIF-2α share significant sequence homology and both are regulated post-translationally by protein degradation [19], [20]. HIF-2α, originally termed endothelial PAS domain protein 1 (EPAS1) because of its expression in endothelial cells, exhibits a more restricted expression pattern than HIF-1α [17], [22]. Although both HIF-α subunits are able to bind the consensus hypoxia-responsive element (HRE) in promoters that contain the sequence NCGTG, they seem to regulate a different set of target genes depending on the cellular context and oxygen concentration [23], [24]. The factors and molecular mechanisms that potentially determine this isoform-specific target gene selectivity remain poorly defined. Interestingly, although HIF-1α was originally identified to bind to HRE in the 3′-enhancer of the EPO gene, there is now considerable evidence that HIF-2α is the main HIF-α-subunit controlling EPO gene expression both *in vitro* and *in vivo* [25].

We previously reported that the volatile anesthetic halothane inhibits hypoxia-induced activation of HIF-1 by distinct molecular mechanisms [26]. Recently, however, another volatile anesthetic, isoflurane, has been reported to upregulate HIF-1 activity in Hep3B cells [27], cultured rat hippocampal neurons [28], and rat myocardium [29]. Isoflurane-induced activation of HIF is now considered a possible mechanism of anesthetic preconditioning [27], [28], [29], [30], [31]. However, *in vivo* experiments of the brain have not been reported, and while HIF-2 rather than HIF-1 mainly regulates EPO in the brain [32], the effect of general anesthetics on HIF-2 has not been well investigated. Considering the pivotal role of EPO in inducing neuroprotection, the influence of general anesthetics on EPO, especially in the brain, may have a major impact on perioperative clinical management. In the present study, we investigated the effect of general anesthetics, including isoflurane, on hypoxia-induced upregulation of EPO in the mouse brain and primary cultured astrocytes.

## Results

### Isoflurane inhibits the induction of EPO expression under hypoxic conditions in the brain, but does not affect EPO induction in the kidney

**Figure 1:**
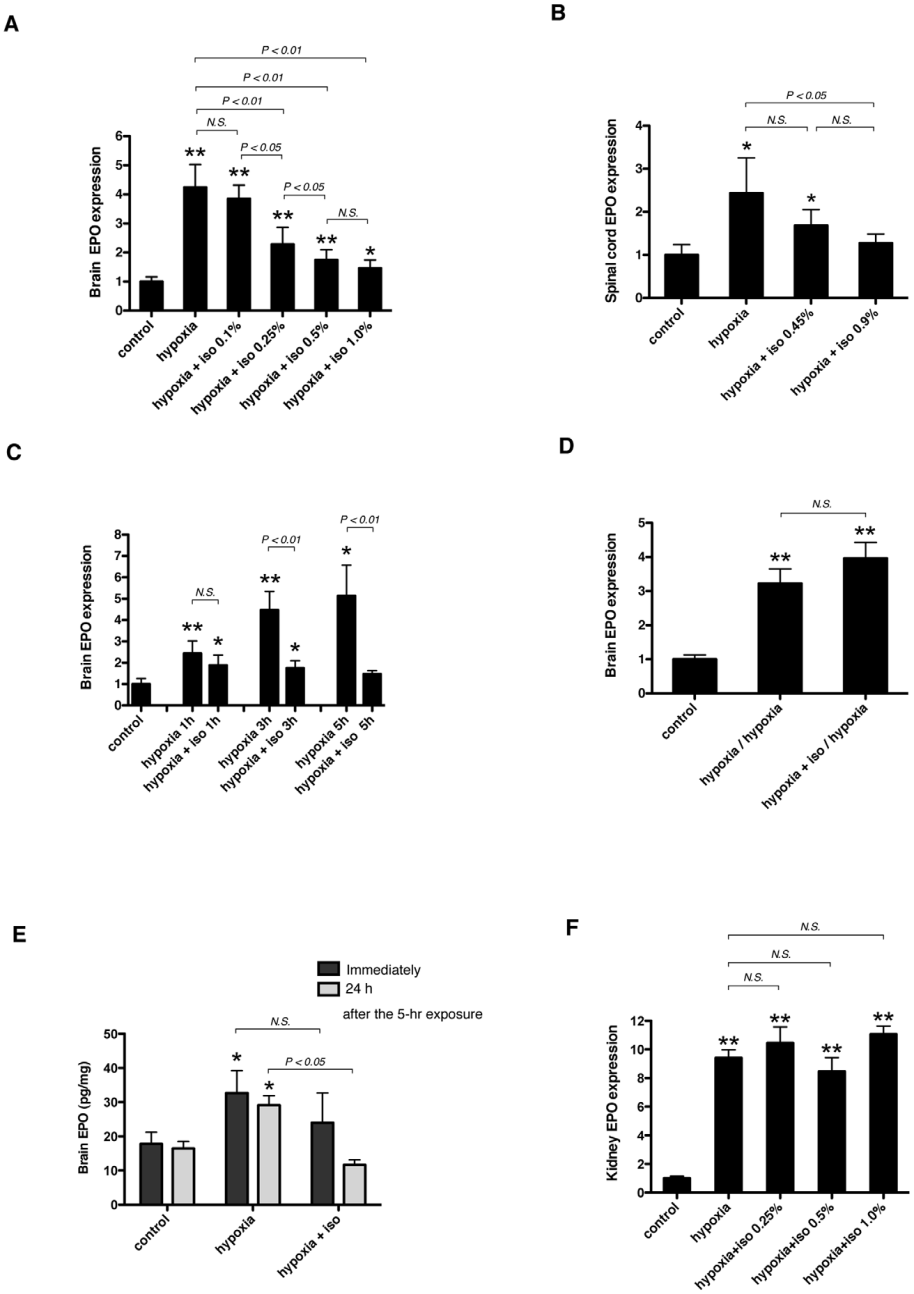
Effect of isoflurane on EPO expression in mouse CNS and kidney. (A, B, and F) Six-week-old BALB/c mice were exposed to 10% O_2_ (hypoxia) in the presence of various concentrations of isoflurane for 3 hours (n = 6–15), or (C) exposed to 10% O_2_ (hypoxia) with 0.5% isoflurane for the indicated periods of time. (D) 24 hours after the hypoxic exposure with or without 0.5% isoflurane, 6-week-old BALB/c mice were re-exposed to hypoxia (10% O_2_) for 3 hours. EPO mRNA in the brain (A, C, and D), spinal cord (B) and kidney (F) was assayed with real-time RT-PCR analysis. (E) Immediately or 24 hours after the 5-hour hypoxic (10% O_2_) exposure with or without 0.5% isoflurane, EPO protein concentration (pg/ml) in the brain was quantified with ELISA and divided by the total protein concentration (mg/ml) of each mouse brain. Number of animals per treatment conditions is 6 (C–E). Data are presented as mean ± SD. The expression levels of EPO were normalized to that of 18S and expressed relative to the mean of control mice (A, B, C, D and F). *P<0.05, **P<0.01 versus control, *N.S.*; not significant (Mann-Whitney U-test).

To examine the effect of general anesthetics on EPO expression under hypoxic conditions, we exposed 6-week-old BALB/c mice to 10% O_2_ (hypoxia) for 3 h with isoflurane. Hypoxic exposure significantly increased EPO mRNA expression and isoflurane suppressed hypoxia-induced EPO mRNA expression in a concentration-dependent manner in the mouse brain (Figure 1A) and spinal cord (Figure 1B). In the brain, a decrease was observed at 0.25% and maximal suppression was achieved at 0.5% (Figure 1A). Next, we performed a time course study with 0.5% isoflurane. Hypoxic exposure increased EPO mRNA time-dependently (Figure 1C). Isoflurane inhalation for more than 1 h significantly suppressed induction (Figure 1C). We re-exposed mice to hypoxia 24 h after hypoxic treatment with (hypoxia+iso/hypoxia) or without (hypoxia/hypoxia) 0.5% isoflurane to determine the reversibility of the suppressive effect of isoflurane on EPO mRNA in the brain. As shown in Figure 1D, hypoxia-induced upregulation of EPO mRNA was almost of a similar extent between the two groups (hypoxia/hypoxia and hypoxia+iso/hypoxia); therefore, the suppressive effect of isoflurane was not identified 24 h later. Next, in order to confirm the effect of isoflurane at the protein level, we measured EPO protein concentration in mouse brains using ELISA. We exposed mice to 10% O_2_ (hypoxia) for 5 h with or without 0.5% isoflurane. Hypoxic exposure induced a significant increase in EPO protein immediately after the treatment, and the effect of isoflurane was not apparent (Figure 1E). However, 24 h after the hypoxic exposure, EPO protein concentration decreased in the mice exposed to hypoxia with isoflurane, although still elevated in the mice without isoflurane (Figure 1E). Hypoxia has been reported to induce EPO mRNA upregulation in the kidney and brain [30]. Therefore, we measured EPO mRNA levels in the kidney. Hypoxic exposure significantly increased EPO mRNA in the kidney, but isoflurane did not affect the induction of EPO mRNA levels (Figure 1F).

### Sevoflurane, halothane, nitrous oxide (N_2_O), pentobarbital, ketamine,and propofol suppress hypoxia-induced up-regulation of EPO mRNA in mouse brains

**Figure 2:**
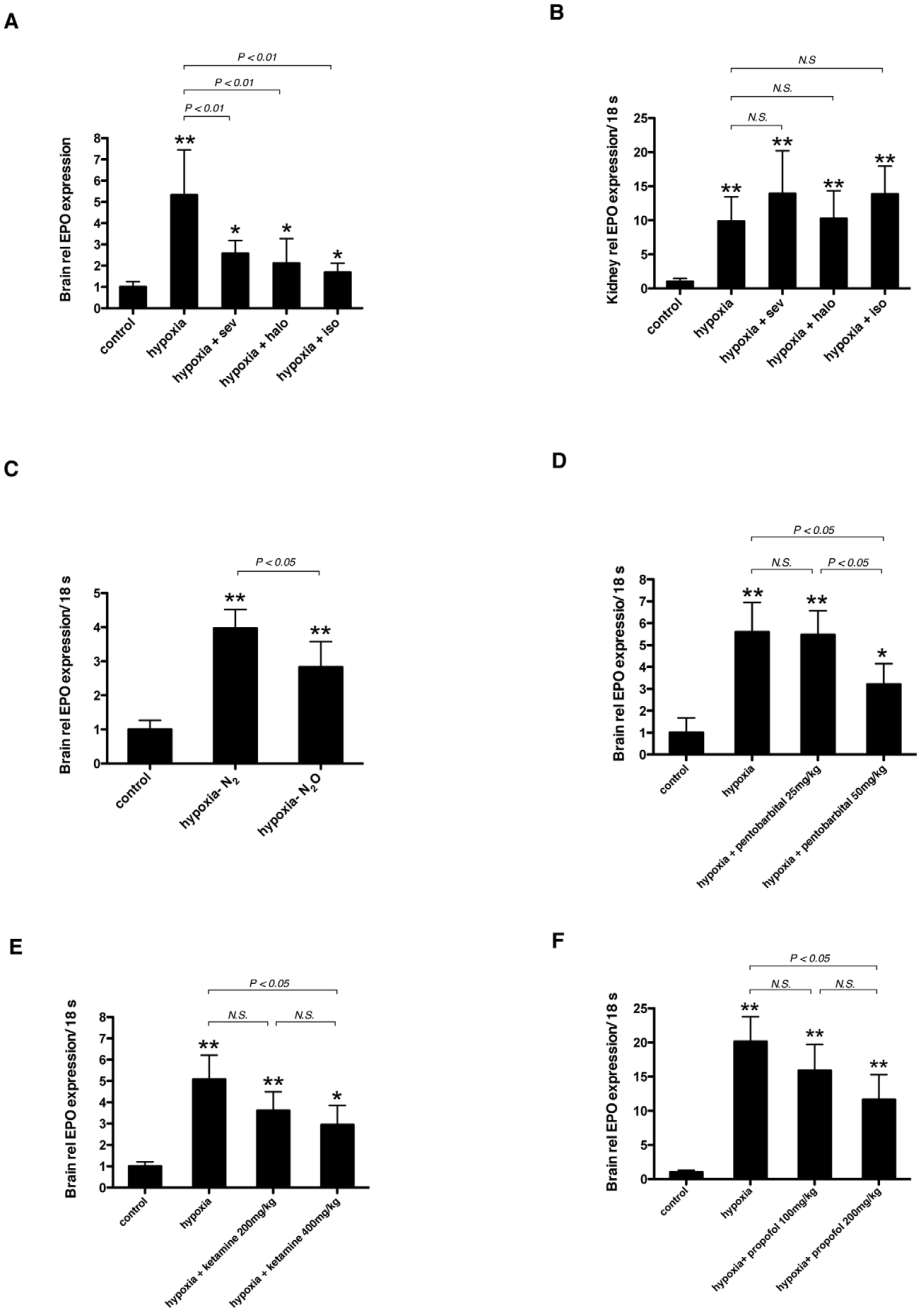
Effect of various anesthetics on hypoxia-induced EPO upregulation in the brain. (A, B) 6-week-old BALB/c mice were exposed to 10% O_2_ (hypoxia) in the presence of 0.5% sevoflurane, halothane or isoflurane for 3 hours. (C) 6-week-old mice were exposed to 10% O_2_, 90% N_2_O (hypoxia- N_2_O) for 3 hours and compared with 10% O_2_, 90% N_2_ (hypoxia- N_2_). (D–F) 6-week-old mice were exposed to 10% O_2_ with pentobarbital (D), ketamine (E) or propofol (F). In the all experiments, control mice were exposed to air without anesthetics (normoxia). EPO mRNA in the brain (A, C–F) and kidney (B) was assayed with real-time RT-PCR. Data are presented as mean ± SD (n = 6). The expression levels of EPO were normalized to that of 18S and expressed relative to the mean of control mice. *P<0.05, **P<0.01 versus control, *N.S.*; not significant (Mann-Whitney U-test).

To examine whether the effect of isoflurane could be observed with other general anesthetics, we exposed 6-week-old BALB/c mice to 10% O_2_ with 0.5% sevoflurane or halothane. As in the case of isoflurane, both anesthetics also suppressed hypoxia-induced EPO upregulation in the brain (Figure 2A) but did not affect the expression of EPO in the kidney (Figure 2B). Next, we investigated the effect of nitrous oxide (N_2_O). We exposed mice to 10% O_2_ and 90% N_2_O (hypoxia-N_2_O group), compared with 10% O_2_ and 90% N_2_ (hypoxia-N_2_ group). EPO mRNA in the brains of the hypoxia-N_2_O group was significantly suppressed compared with that in the hypoxia-N_2_ group (Figure 2C). Finally, we tested the non-inhalational anesthetics pentobarbital, ketamine, and propofol. Fifty mg/kg pentobarbital (Figure 2D), 400 mg/kg ketamine (Figure 2E), and 200 mg/kg propofol (Figure 2F) also suppressed hypoxia-induced EPO mRNA upregulation in the brain.

### Isoflurane inhibits the induction of EPO expression under hypoxic conditions in the brain of one-week and sixteen-week old C57BL/6N CrSlc mice

**Figure 3:**
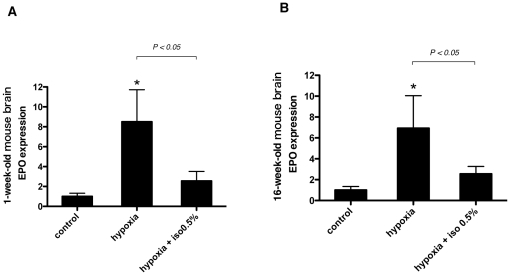
Effect of age and species on hypoxic EPO induction in mice brains. (A) 1-week and (B) 16-week-old C57BL/6N CrSlc mice were exposed to 10% O_2_ (hypoxia) in the presence of 0.5% isoflurane for 3 hours. Control mice were exposed to air without anesthetics (normoxia). EPO mRNA in the brain was assayed with real-time RT-PCR. Data are presented as mean ± SD (n = 3–5). The expression levels of EPO were normalized to that of 18S and expressed relative to the mean of control mice. *P<0.05 versus control (Mann-Whitney U-test).

Age is an important factor that influences the response to hypoxia-ischemia in the brain [33]. After our first experiment, we performed the same experiment in mice of other ages and species. One-week and sixteen-week-old C57BL/6N CrSlc mice were exposed to 10% O_2_ (hypoxia) for 3 h with 0.5% isoflurane. As with 6-week-old BALB/c mice, EPO mRNA induction was significantly suppressed with isoflurane in one-week (Figure 3A) and sixteen-week (Figure 3B) old mice.

### Systemic hemodynamics

To exclude the possibility of secondary effects, including hypotension, influencing the brain's hypoxic responses, we examined the systemic hemodynamics of mice. Hemodynamic parameters including heart rate (HR), systolic (SAP), diastolic (DAP), and mean (MAP) arterial pressures were measured in 6-week-old BALB/c mice exposed to 10% O_2_ (hypoxia), 10% O_2_ with 0.5% isoflurane (hypoxia+iso), or 10% O_2_ with 0.5% sevoflurane (hypoxia+sev) for 3 h, compared with controls (Table 1). Control mice were exposed to air without anesthetics. SAP and MAP decreased in the hypoxia, hypoxia+iso, and hypoxia+sev groups compared to the control group. However, there were no significant differences in all hemodynamic parameters among the hypoxia, hypoxia+iso, and hypoxia+sev groups.

**Table 1:** Systemic hemodynamics.

Mice	n	HR, beats/min	SAP, mmHg	DAP, mmHg	MAP, mmHg
Control	7	450±70	115±13	62±15	80±12
Hypoxia	7	513±49	100±5.8*	50±17	67±12*
Hypoxia+Iso	6	463±77	93±8.8*	42±7.3	58±5.6*
Hypoxia+Sev	6	448±34	89±8.7*	54±12	65±10*

Six-week-old BALB/c mice were divided into 4 groups; control (exposed to air), hypoxia (exposed to 10% O_2_ for 3 hours), hypoxia+iso (exposed to 10% O_2_ with 0.5% isoflurane for 3 hours) and hypoxia+sev (exposed to 10% O_2_ with 0.5% sevoglurane for 3 hours). Immediately after the hypoxic exposure, heart rate and blood pressure were measured.

There were no significant differences in any of the parameters among hypoxia, hypoxia+iso and hypoxia+sev group mice. Values are shown as mean ± SD. * *P*<0.05 versus control.

HR = heart rate; SAP = systolic arterial pressure; DAP = diastolic arterial pressure; MAP = mean arterial pressur.

### Isoflurane inhibits the induction of HIF-2α protein expression under hypoxic conditions in the brain of mice

**Figure 4:**
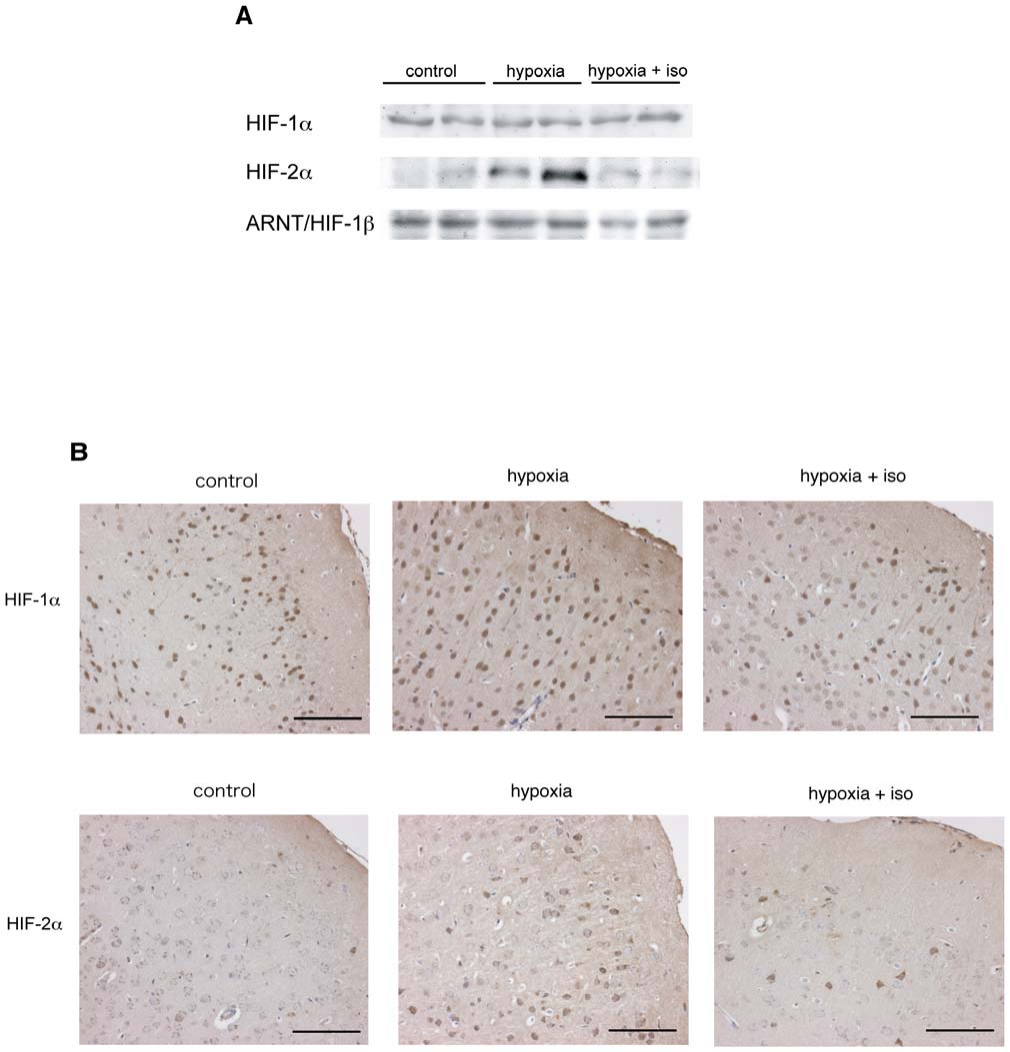
Mechanism of suppression by isoflurane against EPO upregulation under hypoxic conditions. (A) Expression analysis of hypoxia-inducible factor (HIF)-1α, HIF-2α, and aryl hydrocarbon receptor nuclear translocator (ARNT, also termed as HIF-1β) in the whole brain by immunoblotting. 6-week-old BALB/c mice were exposed to air (control), 10% O_2_ (hypoxia) or 10% O_2_ with 0.5% isoflurane (hypoxia+iso) for 3 hours. Figures are representative of at least three independent experiments. (B) Immunohistochemical staining for HIF-1α and HIF-2α in the frontal cortex of 6-week-old BALB/c mice. Mice were exposed to air (control), 10% O_2_ (hypoxia) or 10% O_2_ with 0.5% isoflurane (hypoxia+iso) for 3 hours. Figures are representative of 6 slices of 3 mice. Scale bars: 100 µm.

EPO is induced under hypoxic conditions, mainly through activation of HIF-1 and HIF-2 [16], [34]. Therefore, we investigated the effect of isoflurane on the expression of HIF-1α and HIF-2α proteins. We exposed 6-week-old BALB/c mice to 10% O_2_ (hypoxia) for 3 h with or without 0.5% isoflurane. Control mice were exposed to air without isoflurane (normoxia). The protein expression of HIF-1α, HIF-2α, and ARNT (HIF-1β) was investigated with an immunoblot assay. As shown in Figure 4A, HIF-1α protein was expressed under normoxic conditions and this expression was not significantly altered in response to either hypoxic exposure or isoflurane. In contrast, hypoxic exposure induced a marked accumulation of HIF-2α protein, which was clearly suppressed by isoflurane (Figure 4A). ARNT protein expression was almost stable under all conditions (Figure 4A). Next we assayed the expression of HIF-1α and HIF-2α immunohistochemically, and found positive immunostaining of HIF-1α was observed globally in the frontal cortex under all conditions (Figure 4B). HIF-2α was also admitted to a certain degree under hypoxic condition, but barely observed under normoxia or hypoxia with isoflurane exposure (Figure 4B).

### Effect of isoflurane on the expression of HIF target genes

**Figure 5:**
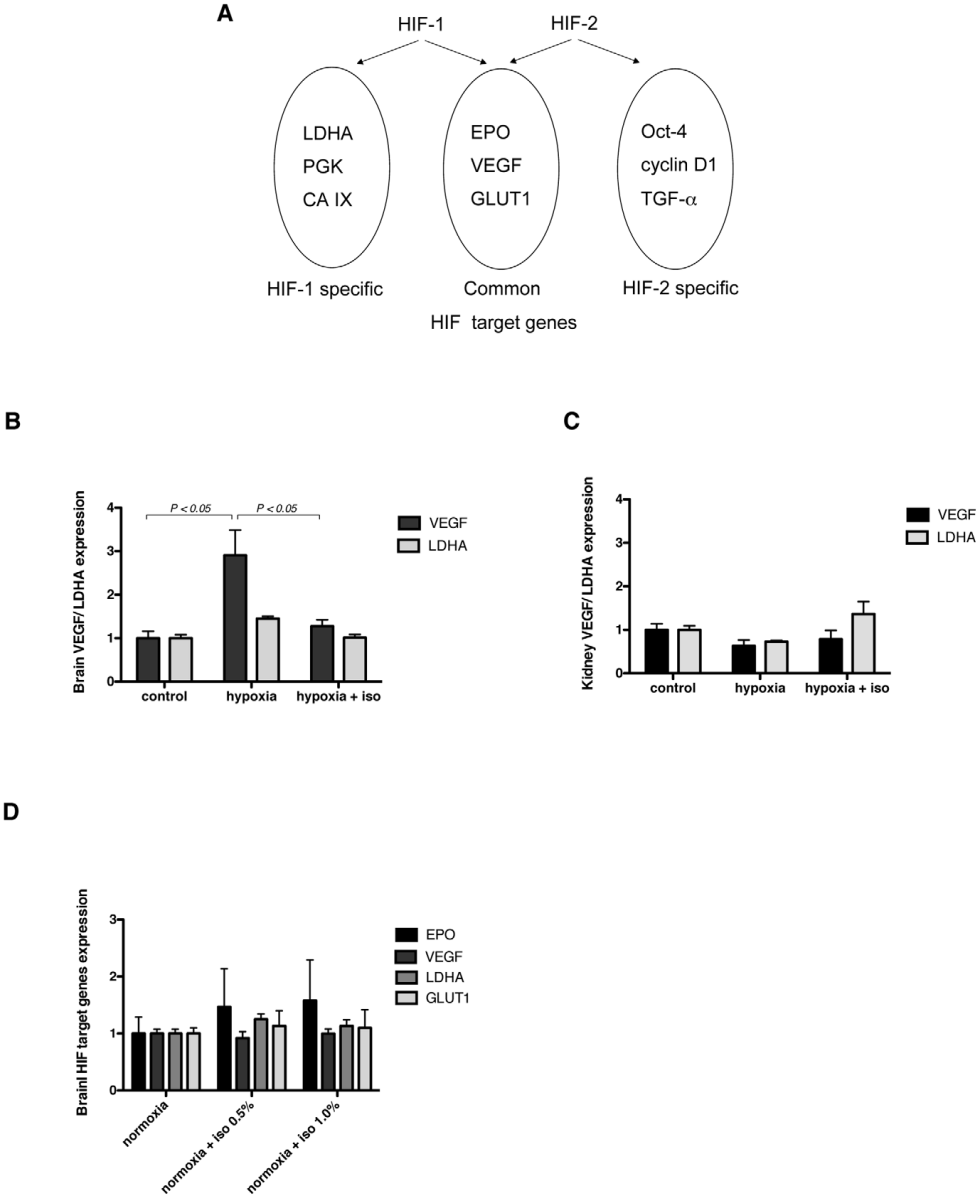
Effect of isoflurne on mRNA expression of HIF target genes. (A) HIF-1 and HIF-2 have unique, as well as common, target genes. HIF-1 specifically regulates glycolytic genes, including lactate dehydrogenase A (LDHA), phosphoglycerate kinase (PGK), as well as carbonic hydrase-9 (CA IX) whereas HIF-2 exclusively regulates POU transcription factor Oct-4, cyclin D1, and transforming growth factor α (TGF-α). Other hypoxia-inducible genes, such as vascular endothelial growth factor (VEGF), glucose transporter 1 (GLUT1), and EPO are regulated by both HIF-1 and HIF-2. (B, C) 6-week-old BALB/c mice were exposed to 10% O_2_ (hypoxia) for 3 hours with or without 0.5% isoflurane and compared with controls. Control mice were exposed to air without isoflurane (normoxia). (D) 6-week-old BALB/c mice were exposed to 0.5% or 1.0% isoflurane in air for 3 hours. Data are presented as mean ± SD (n = 6). The expression levels of EPO, VEGF, LDHA and GLUT1 were assayed using real-time RT-PCR and normalized to that of 18S and expressed relative to the mean of mice exposed to air without isoflurane (normoxia).

Despite significant similarities in their DNA binding and dimerization domains, it has been demonstrated that HIF-1 and HIF-2 have unique, as well as common, target genes [35]. As shown in Figure 5A, HIF-1 specifically regulates glycolytic genes, including lactate dehydrogenase A (LDHA), whereas HIF-2 exclusively regulates POU transcription factor Oct-4, cyclin D1, and transforming growth factor α (TGF-α) [35], [36]. Other hypoxia-inducible genes, such as vascular endothelial growth factor (VEGF), glucose transporter 1 (GLUT1), and EPO, are regulated by both HIF-1 and HIF-2 in a cell-type-specific manner [35]. Therefore, we investigated the influence of hypoxia or isoflurane on HIF target genes in the mouse brain. VEGF mRNA, as well as EPO, was significantly induced by hypoxic exposure (10% O_2_, 3 h) and was significantly suppressed with isoflurane (0.5%) (Figure 5B). In contrast, the expression of LDHA did not change significantly with hypoxic exposure and isoflurane inhalation (Figure 5B). Next, we studied the change of HIF target genes in the kidney. Although hypoxic exposure significantly induced EPO mRNA in the kidney (Figure 1F), other HIF target genes, VEGF and LDHA, were not elevated but rather suppressed (Figure 5C). The effect of isoflurane on VEGF and LDHA was not significant (Figure 5C).

Recently, several reports have shown that isoflurane activates HIF-1 and upregulates HIF target genes [27], [28], [29], [31]. Most of these studies were performed under normoxic conditions. Therefore, we investigated the influence of isoflurane on the expression of HIF target genes in mouse brains under normoxic conditions. We exposed mice to air with 0.5% or 1.0% isoflurane for 3 h and measured the mRNA levels of the HIF target genes, including EPO, VEGF, LDHA, and GLUT1 (Figure 5D). Isoflurane inhalation did not significantly change the expression of the mRNA for any of these HIF target genes.

### Isoflurane, pentobarbital, ketamine, and propofol suppress, but morphine does not suppress hypoxia-induced up-regulation of EPO mRNA in primary cultured astrocytes

**Figure 6:**
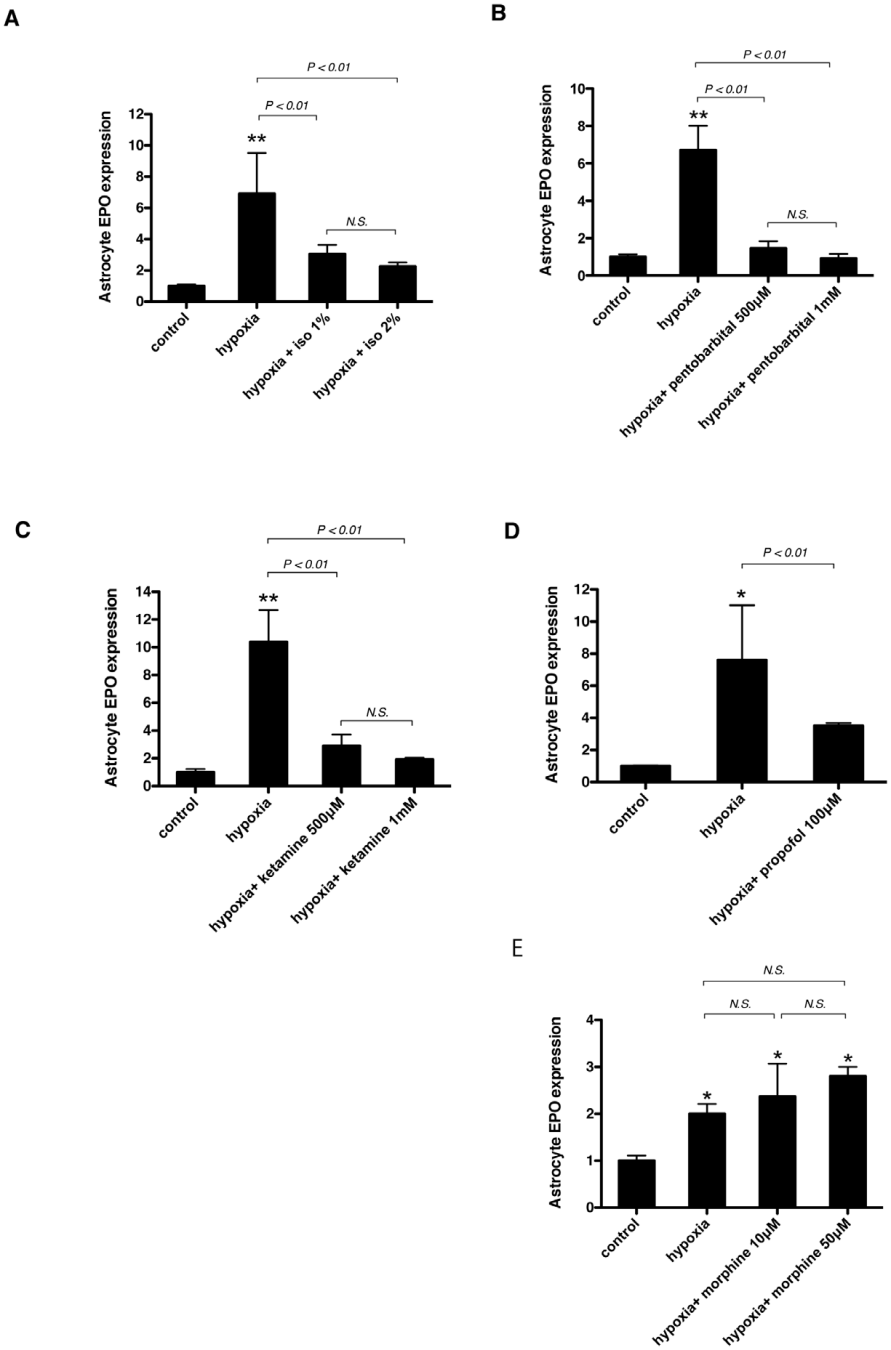
Effect of various anesthetics on EPO expression in primary cultured astrocytes. Primary cultured astrocytes were exposed to 1% O_2_ (hypoxia) in the presence of indicated concentrations of isoflurane (A), pentobarbital (B), ketamine (C), propofol (D) or morphine (E) for 4 hours. In the all experiments, control was exposed to 20% O_2_. EPO mRNA was assayed with real-time RT-PCR. Data are presented as mean ± SD (n = 4). The expression levels of EPO were normalized to that of 18S and expressed relative to the mean of control. *P<0.05, **P<0.01 versus control, *N.S.*; not significant (Mann-Whitney U-test).

**Figure 7:**
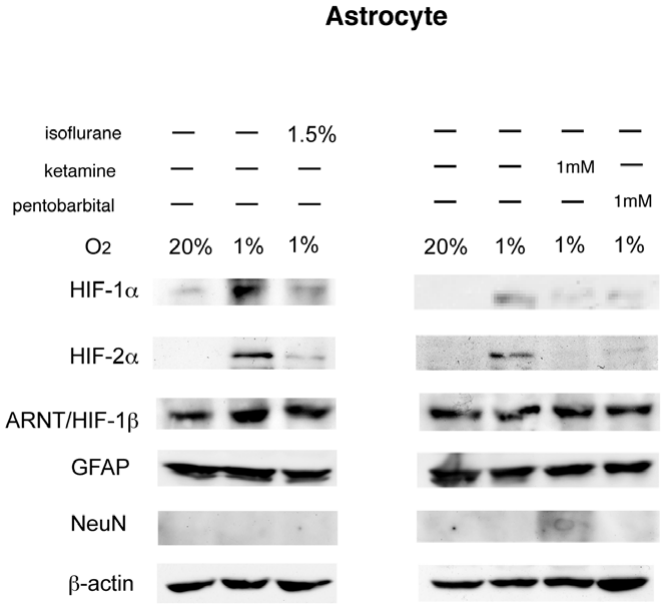
Expression analysis of HIF-1α, HIF-2α, and ARNT protein in primary cultured astrocytes by immunoblotting. Primary cultured astrocytes were incubated under hypoxic (1% O_2_) conditions with or without 1.5% isoflurane, 1 mM pentobarbital, or 1 mM ketamine for 4 hours. Whole cell lysates were analyzed for HIF-1α, HIF-2α, ARNT, GFAP, NeuN and β-actin protein expression by immunoblot assay. Figures are representative of at least three independent experiments.

Astrocytes are now supposed to be the major source of EPO in the CNS [6], [7]. To investigate the direct effect of general anesthetics on astrocytes, we performed *in vitro* experiments. Primary cultured astrocytes were exposed to hypoxia (1% O_2_) with or without various anesthetics for 4 h. Hypoxic exposure significantly induced EPO mRNA, and isoflurane suppressed its induction (Figure 6A). Not only isoflurane but also other anesthetics, including pentobarbital, ketamine, and propofol, suppressed the induction of EPO mRNA (Figures 6B–D). However, morphine, a commonly used drug during the preoperative period, did not inhibit the upregulation of EPO mRNA in astrocytes (Figure 6E). Next, HIF-1α, HIF-2α, and ARNT protein accumulation were analyzed in whole cell lysates from astrocytes. An immunoblot assay showed distinct HIF-1α and HIF-2α protein accumulation under hypoxic conditions, and the expression was significantly suppressed with isoflurane, ketamine, and pentobarbital (Figure 7).

### Effect of various anesthetics on oxygen consumption in primary cultured astrocytes

**Figure 8:**
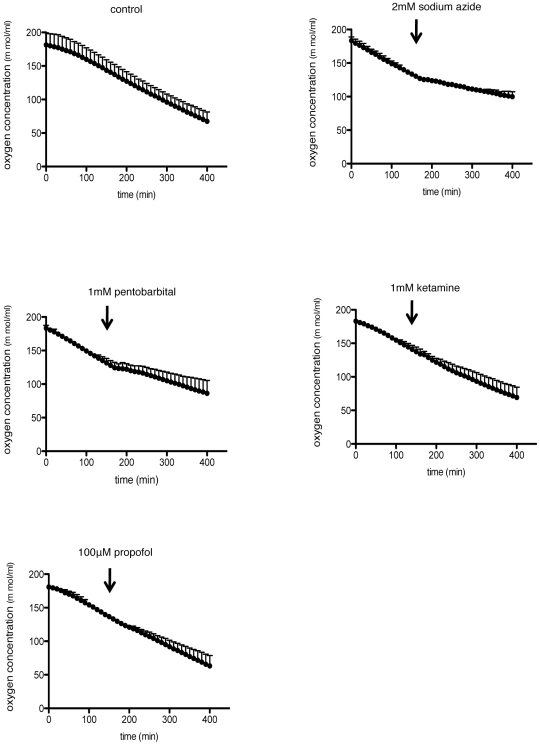
Effect of anesthetics on oxygen consumption of primary cultured astrocytes. Oxygen consumption curves generated using a Clark electrode for primary cultured astrocytes suspensions. Arrows indicate addition of 2 mM sodium azide, 1 mM pentobarbital, 1 mM ketamine or 100 µM propofol. The slope of the curve is a measure of the rate of O_2_ consumption. Data are presented as mean ± SD (n = 3).

To investigate the precise mechanism of how general anesthetics suppress the induction of EPO under hypoxic conditions, we finally examined the influence of general anesthetics on oxygen consumption in astrocytes. General anesthetics, especially thiopental, are known to decrease metabolism and oxygen consumption of the brain [37]. Therefore, the suppression of oxygen consumption induced by general anesthetics may reduce the level of hypoxia and consequently decrease HIF-α protein accumulation and EPO induction. As indicated in Figure 8, 1 mM pentobarbital as well as sodium azide, a cytochrome oxidase inhibitor, reduced the oxygen consumption; however, 1 mM ketamine and 100 µM propofol did not alter the oxygen consumption significantly. These results suggest that, at least as to ketamine and propofol, the decrease in oxygen consumption was not the cause of the suppression of EPO induction.

## Discussion

EPO is a major hematopoietic growth factor that is mainly produced in the kidney and fetal liver [5]. It is also known to express in CNS tissue [5]. EPO mRNA is constitutively expressed in the cortex and hippocampus of the brain [38]. Various studies have focused on the function of EPO in CNS; for example, mice lacking EPO or EPOR exhibited increased apoptosis in the brain before they died from severe anemia *in utero* [39], [40], and mice lacking EPOR in the brain suffered from reduced neurogenesis or impaired migration of neurons in a brain stroke model [41]. Thus, EPO is considered to be a neuroprotective factor against hypoxic-ischemic and traumatic injuries and essential for neuronal development [5], [12].

In the present study, we showed that the induction of EPO expression under hypoxic conditions was suppressed by the general anesthetic isoflurane in a concentration- and time-dependent manner in the mouse brain. Other anesthetics, including sevoflurane, halothane, N_2_O, pentobarbital, ketamine, and propofol, showed a similar effect. As for the mechanism of this suppression, we found that the accumulation of HIF-2α, but not HIF-1α, protein under hypoxic conditions was suppressed with isoflurane in the mouse brain. This finding is consistent with a previous report indicating that EPO is a target gene for HIF-2α, rather than HIF-1α, in CNS [32]. HIF-1α is expressed ubiquitously, but the expression of HIF-2α is tissue-specific [32]. HIF-2α is expressed in astrocytes and endothelial cells in the CNS [32]. Astrocytes are the major source of EPO in the CNS [6], [7], and hypoxia-induced EPO upregulation is dramatically reduced in the astrocyte-specific HIF-2α knockout mouse [38]. In the present study, various anesthetics, including isoflurane, pentobarbital, and ketamine, suppressed the accumulation of HIF-2α protein under hypoxic conditions in cultured astrocytes. Therefore, our results indicated that the hypoxia-induced activation of HIF-2 in astrocytes was inhibited by general anesthetics, which resulted in a significant suppression of EPO production.

Recently, various studies on astrocytes have been performed, and these cells are considered responsible for a wide variety of functions in the CNS, including synaptic transmission and information processing by neural circuit functions [42]. In the present study, we showed that various anesthetics suppressed the accumulation of HIF-2α protein and EPO upregulation under hypoxic conditions in the mouse brain and cultured astrocytes. Considering the fact that the accumulation of HIFα proteins is induced by hypoxia, the suppression of oxygen consumption induced by general anesthetics may reduce the level of hypoxia and consequently decrease HIF-2α protein accumulation. Actually, most of all general anesthetics excluding ketamine and N_2_O are known to decrease cerebral metabolic rate of oxygen [43]. But the effect of anesthetics on metabolism of astrocytes is not well investigated. In the current study, pentobarbital, well known for suppressing the metabolism of the CNS [37], decreased oxygen consumption of astrocytes. On the other hand, ketamine and propofol did not change oxygen consumption. In addition, we previously reported that hypoxic brain EPO induction was preserved in hypothermic mice, although hypothermia is well known to reduce cerebral oxygen consumption [44]. Therefore, these findings suggest that the suppressive effect of various anesthetics against HIF-2α protein accumulation and EPO induction cannot be explained only by the decrease in oxygen consumption.

The main target of general anesthetics differs with various anesthetics; for example, ketamine and N_2_O act via N-methyl-D-aspartate (NMDA) receptors [45], [46], whereas the volatile anesthetics, propofol and barbiturates act via γ-aminobenzoic acid-A (GABA-A) receptors [47], [48]. On the other hand, the effect of general anesthetics on glial cells is not well understood, except for the fact that volatile anesthetics inhibit the glutamate uptake of astrocytes [49]. Our finding that various general anesthetics have an EPO-suppressive effect in *in vitro* experiments suggests that general anesthetics have some common direct effects on astrocytes. This finding is quite surprising considering the diverse action mechanism of general anesthetics. Anesthetics modulate functions of macromolecules, which play an essential role in cellular signal transduction. For example, protein kinase C (PKC) [50], mitogen-activating protein kinases (MAPKs) [51], and reactive oxygen species (ROS) [52] are modulated by anesthetics. PKC, MAPK, and ROS are also identified to affect HIF activity by modulating HIF-α protein translation rate, hydroxylation, and phosphorylation of HIF-α protein [53], [54], [55]. Therefore, general anesthetics may affect astrocytes through modulation of such enzymes and mediators. But most of the studies considering the effect of anesthetics on HIF have focused on HIF-1 under normoxic conditions, and the effect on HIF-2 under hypoxic conditions is not well understood.

Another important finding of the present study is the difference of behavior between HIF-1α and HIF-2α. Namely, HIF-1α was expressed even under normoxic conditions and 3-h hypoxic exposure did not affect HIF-1 protein accumulation distinctively in mice brains. In contrast, HIF-2α was barely expressed under normoxic conditions and clearly increased in response to hypoxic exposure. In *in vitro* experiments, however, both HIF-1α and HIF-2α protein accumulation were observed under a 1% O_2_ condition, and various anesthetics significantly suppressed their induction. Although both HIF-1α and HIF-2α are considered to accumulate significantly under hypoxic conditions in *in vivo* experiments using the mouse brain [56], some reports have shown that HIF-1α was expressed even under normoxic conditions [57], [58]. In most of these previous reports, HIF-1α protein increased in response to hypoxic exposure in the brain, but the extent varied [57], [58], [59]. A possible explanation for the discrepancy is the difference of oxygen concentration. Previous report showed that HIF-1α protein accumulation was observed under 1% O_2_ condition but not 5% O_2_ condition in the neuronal cell line SK-N-BE cells [23]. We exposed mice to 10% O_2_ in our studies, but 10% O_2_ might not be low enough to induce HIF-1α protein accumulation.

EPO has now been considered to be one of the promising agents for neuroprotection [3], [4], [5]. Actually, in the clinical trials, erythropoietin showed neuroprotective effect against acute stroke [60], hypoxic-ischemic encephalopathy in newborns [61] and delayed ischemic deficits following aneurysmal subarachnoid hemorrhage [62]. In the current study, we showed the induction of EPO mRNA expression under hypoxic conditions was suppressed with isoflurane in a concentration- and time-dependent manner. Other anesthetics including sevoflurane, halothane, N_2_O, pentobarbital, propofol and ketamine showed the same effect. Most of all anesthetics suppressed EPO mRNA induction with concentrations no more than clinically used, for example, isoflurane, sevoflurane and halothane showed this effect with 0.5%. Therefore, considering the neuroprotective effect of EPO, exposure to anesthetics beyond necessity should be avoided especially in cases hypoxia in brain may happen at greater risk like cardiovascular surgery.

According to the recent reports, anesthetic exposure in neonatal animals leads to neuronal death in certain circumstances [63], [64]. Such neurotoxicity has now been demonstrated for many anesthetics, including isoflurane, ketamine, midazolam, pentobarbital, N_2_O, and propofol, and a positive correlation may exist between increased levels of anesthesia and increased severity of neuroapoptosis [65], [66]. The precise mechanisms by which injury is invoked are not clear, although an imbalance between excitatory and inhibitory input in the CNS during synaptogenesis may contribute to such an effect [65]. On the other hand, EPOR is highly expressed in the developing mouse brain [7], and mice lacking EPO or EPOR experienced increased apoptosis in the brain before they died of severe anemia in the uterus [39], [40]. We did not investigate the effect of general anesthetics on brain EPO under normoxic conditions in neonatal animals. However, considering the pivotal role of EPO in brain development, general anesthetics may cause neuroapoptosis by suppressing EPO production in the brain. Further studies using neonatal animals should be performed.

In conclusion, we demonstrated that isoflurane inhibited hypoxia-induced EPO upregulation in the mouse brain and cultured astrocytes, most likely through suppression of HIF-2 activity. Other general anesthetics showed the same effect. Our findings suggest that general anesthetics have some direct effect on astrocytes and a major impact on the hypoxic response of the CNS.

## Methods

### Animals

This study (ID: Med Kyo 09504) was approved by the Animal Research Committee of Kyoto University (Kyoto, Japan), and all experiments were conducted in accordance with the institutional and NIH guidelines for the care and use of laboratory animals. All procedures were performed on BALB/c or C57BL/6N CrSlc mice purchased from Japan SLC Inc., Shizuoka, Japan. Food and water were provided *ad libitum*, and the mice were maintained under controlled environmental conditions (24°C, 12-h light/dark cycles).

### Drugs and chemicals

Isoflurane and pentobarbital were obtained from Dainippon Sumitomo Pharma Co., Ltd., Osaka, Japan; sevoflurane from Maruishi Pharmaceutical Co., Ltd., Osaka, Japan; halothane from Takeda Pharmaceutical Co., Ltd., Osaka, Japan; and propofol from Astra-Zeneca, London, UK. Morphine and ketamine were purchased from Sankyo Co., Ltd., Tokyo, Japan. Nitrous oxide (N_2_O) (Wakayama Sanso, Wakayama, Japan), oxygen (O_2_) (Taiyo Nippon Sanso, Tokyo, Japan), and nitrogen (N_2_) (Taiyo Nippon Sanso) were also used.

### Hypoxic treatment

Mice were placed in a polypropylene chamber, and O_2_ and N_2_ mixed gas with or without volatile anesthetics, including isoflurane, sevoflurane, and halothane, was delivered to the chamber at a flow rate of 3 l/min using an anesthetic machine (Custom50; Aika, Tokyo, Japan). In the experiment using N_2_O, O_2_ and N_2_O-mixed gas was administered at the same flow rate. Concentrations of O_2_, carbon dioxide (CO_2_), N_2_O, and volatile anesthetics, including isoflurane, sevoflurane, and halothane, were monitored continuously using an infrared analyzer (Capnomac Ultima; Datex-Ohmeda, Helsinki, Finland). Mice were allowed to adjust to the hypoxic environment by gradually decreasing the O_2_ level from 21% to 10% over 1 h, and they were maintained at 10% O_2_ for the indicated durations. Treatment with the volatile anesthetics was initiated immediately after the adaptation to hypoxia. In the experiments using pentobarbital, ketamine, and propofol, the drugs were administered intraperitoneally immediately after hypoxic adaptation. The rectal temperature was monitored using an ATB-1100 (Nihon Kohden, Tokyo, Japan), and a heat lamp was used to maintain the temperature at 36±1°C. Arterial blood pressure was measured non-invasively using the tail-cuff method immediately after completion of the hypoxic exposure using an MK-2000ST (Muromachi Kikai Co., Ltd., Tokyo, Japan). At the end of the experiments, the mice were killed by cervical dislocation. The brains, spinal cords, and kidneys were rapidly removed, frozen in liquid nitrogen, and stored at −80°C for subsequent determinations.

### Reverse transcription and real-time quantitative polymerase chain reaction

RNA was isolated from the frontal lobe of the brain using the FastPure™ RNA Kit (Takara Bio, Inc., Shiga, Japan). First-strand synthesis and real-time RT-PCR were performed using the One Step SYBR™ PrimeScript™ RT-PCR Kit II (Takara Bio) according to the manufacturer's instructions. PCR was performed using the Applied Biosystems 7300 Real-Time PCR System (Applied Biosystems, Foster City, CA). All PCR primers (Catalog numbers: 18S: QT01036875; EPO: QT00170331; VEGF: QT00160769; GLUT1: QT01044953) except lactate dehydrogenase A (LDHA) were purchased from Qiagen (Valencia, CA). The sequences of the LDHA primers (Takara Bio) are 5′-GGATGAGCTTGCCCTTGTTGA-3′ (forward) and 5′-GACCAGCTTGGAGTTCGCAGTTA-3′ (reverse). The fold changes in expression of each target mRNA were calculated relative to 18S rRNA.

### ELISA of EPO

Samples were prepared according to the method described previously [67]. Briefly, the entire brain was homogenized in phosphate-buffered saline (PBS), centrifuged for 10 min at 5,000 *g* at 4°C, and immediately frozen at −20°C. After two freeze–thaw cycles to break the cell membranes, the brain homogenates were assayed by an ELISA kit (R&D Systems Europe, Abingdon, UK) according to the manufacturer's instructions. The results were expressed as the ratio of the quantity of EPO (in pg) to the quantity of total protein (in mg) in the brain. The total protein concentration was determined by the modified Bradford assay (Nakalai Tesque, Inc., Kyoto, Japan) using bovine serum albumin (BSA) as a standard.

### Immunoblot assay

Nuclear extracts were prepared from a whole mouse brain using a nuclear extraction kit (Active Motif, Carlsbad, CA). The aliquots (100 µg protein) were fractionated by sodium dodecyl sulfate polyacrylamide gel electrophoresis (SDS/PAGE) (7.5% gel) and subjected to an immunoblot assay following a protocol described previously [68]. Primary antibodies raised against HIF-1α (AB 1536; R&D Systems, Minneapolis, MN), HIF-2α (NB100-480; Novus Biologicals, Inc., Littleton, CO), ARNT (HIF-1β) (#611078; BD Biosciences, San Jose, CA), glial fibrillary acidic protein (GFAP) (#3670; Cell Signaling, Stockholm, Sweden), neuronal nuclei (NeuN) (MAP377; Millipore, Billerica, MA), and β-actin (A5316; Sigma-Aldrich, St. Louis) were used at a 1∶1000 dilution. Horseradish peroxidase (HRP)-conjugated sheep anti-mouse IgG (GE Healthcare, Piscataway, NJ) or donkey anti-rabbit IgG antibodies (GE Healthcare) were also used at a 1∶1000 dilution. The signal was detected with enhanced chemiluminescence (ECL) reagent (GE Healthcare).

### Immunohistochemistry

Immunohistochemistry was performed according to the procedure described by Toda *et al* [69]. Mouse brains were kept at 4°C overnight in 4% paraformaldehyde in 0.1 M phosphate buffer. The brains were then rinsed in PBS, transferred to 70% ethanol, and embedded in paraffin. Ten-micrometer coronal sections were cut and mounted on slides using albumin water. Sections were deparaffinized and rehydrated, and antigen retrieval was performed using autoclaving. Briefly, a Coplin jar containing glass slides in citrate buffer was covered with a loose fitting cap and heated in a stainless steel pressure cooker for 5 min at 121°C. The pressure cooker was removed from the heat source and cooled by running under cold water with the lid on. The glass slides were rinsed in distilled water. The incubation and washing procedures were carried out at room temperature. After deparaffinization and antigen retrieval by the methods noted above, endogenous peroxidase activity was blocked by 0.3% H_2_O_2_ in methyl alcohol for 30 min. The glass slides were washed in PBS (6 times, 5 min each) and mounted with 1% goat normal serum in PBS for 30 min. Subsequently, rabbit polyclonal anti-HIF-1α (AB 1536; R&D Systems) diluted 1∶200 and rabbit polyclonal anti-HIF-2α (NB100-480; Novus Biologicals) diluted 1∶400 were applied overnight at 4°C. They were then incubated with biotinylated goat anti-rabbit serum (second antibody) diluted 1∶300 in PBS for 40 min, followed by washes in PBS (6 times, 5 min each). Avidin-biotin-peroxidase complex (ABC) (ABC-Elite, Vector Laboratories, Burlingame, CA) at a dilution of 1∶100 in BSA was applied for 50 min. After washing in PBS (6 times, 5 min each), coloring reaction was performed using diaminobenzidine, and the nuclei were counterstained with hematoxylin.

### Cell culture

Primary cultures of cerebral cortical astrocytes were prepared from 1- or 2-day-old C57BL/6N CrSlc mice according to the method previously described [70]. Brains of mice were removed under sterile conditions, and the meninges were carefully removed. The tissue was dissociated by passing it through a 320-µm nylon mesh with the aid of a rubber policeman. After washing with Hanks' balanced salt solution containing DNaseI, the cells were suspended and passed through a 100-µm nylon mesh. Next, they were plated on a plastic culture flask (density of 2 brains per flask) in 10-ml tissue culture medium. The tissue culture medium consisted of Dulbecco's modified Eagle's medium (DMEM) supplemented with 10% fetal bovine serum (FBS), 100 U/ml penicillin, and 0.1 mg/ml streptomycin. The cultures were maintained in a humidified atmosphere of 5% CO_2_ in air at 37°C. The medium was changed after 3 days, then twice weekly. At the first medium change, the flasks were vigorously shaken in order to remove oligodendrocytes and their precursors. All experiments were performed in cells at day 14 *in vitro*.

### Isoflurane exposure

Isoflurane exposure was performed as described previously [71]. Briefly, cell dishes were kept in the airtight chamber housed within a water jacket incubator maintained at 37°C. An in-line calibrated anesthetic agent vaporizer was used to deliver isoflurane to the gas phase of the culture wells. Hypoxic gas (1% O_2_–5% CO_2_–94% N_2_) was administered at a flow rate of 3 l/min, until the appropriate effluent concentration of the anesthetic was achieved. Effluent isoflurane, O_2_, and CO_2_ concentrations were continuously monitored via a sampling port connected to an anesthetic agent analyzer (Capnomac Ultima; Datex-Ohmeda, Helsinki, Finland).

### Protein extraction

Whole cell lysates were prepared using ice-cold lysis buffer [0.1% SDS, 1% Nonidet P40 (NP40), 5 mM EDTA, 150 mM NaCl, 50 mM Tris-Cl (pH 8.0), 2 mM DTT, 1 mM sodium orthovanadate, and complete protease inhibitor (Roche Diagnostics)] following a protocol described previously [72]. A total of 100 µg of protein was loaded onto a 7.5% SDS/PAGE gel for immunoblot assay.

### Measurement of total cellular O_2_ consumption

Cells were trypsinized and suspended at 1×10^7^ cells per ml in DMEM with 10% FBS and 25 mM HEPES buffer. For each experiment, equal numbers of cells suspended in 0.4 ml were pipetted into the chamber of an Oxytherm™ electrode unit (Hansatech Instruments, Norfolk, UK), which uses a Clark-type electrode to monitor the dissolved O_2_ concentration in the sealed chamber over time. The data were exported to a computerized chart recorder (Oxygraph; Hansatech Instruments) that calculated the rate of O_2_ consumption. The temperature was maintained at 37°C during measurement. The O_2_ concentration in 0.4 ml of DMEM medium without cells was also measured over time to provide background values. Oxygen consumption experiments were repeated three times.

### Statistical analysis

Data are presented as the mean ± SD. Statistical significance was assessed by Mann-Whitney U-test for two group comparisons and by Kruscal Wallis H-test, followed by Mann-Whitney U-test with Bonferroni Correction for multiple group comparisons. Significance was defined as a value of *P*<0.05.
